# Chronotype in Patients With Immune-Mediated Inflammatory Disease: A Systematic Review

**DOI:** 10.1177/07487304221131114

**Published:** 2022-11-14

**Authors:** Thomas D. Butler, Aala Mohammed Ali, Julie E. Gibbs, John T. McLaughlin

**Affiliations:** * Centre for Biological Timing, Faculty of Biology, Medicine and Health, The University of Manchester, Manchester, UK; † Division of Diabetes, Endocrinology and Gastroenterology, The University of Manchester, Salford Royal NHS Foundation Trust, Manchester Academic Health Science Centre, Manchester, UK

**Keywords:** immune-mediated inflammatory disease, inflammation, circadian, chronotype, systematic review

## Abstract

Immune-mediated inflammatory diseases (IMIDs) such as rheumatoid arthritis, inflammatory bowel disease, and asthma share common pathophysiological pathways characterized by chronic inflammation and subsequent tissue damage involving multiple body sites. Circadian rhythms are 24-h body cycles that regulate immune activity and control the magnitude of immune response based on time of day. Chronotype is a person’s individual circadian phase preference, ranging from morningness to eveningness, which is known to influence the risk of cardiometabolic and mental health disease. We systematically reviewed the literature to assess the association of questionnaire-based chronotype and patients with IMID. A comprehensive search of MEDLINE and Embase identified 12 studies meeting the inclusion criteria, conducted in 7 countries and covering 4 IMIDs to include 15,625 IMID patients and 410,783 healthy controls. Results showed that later chronotype may be a risk factor for worse quality of life and increased symptom burden in patients with IMIDs. In addition, chronotype may be a risk factor for IMID incidence, but the direction and magnitude of this effect were not consistent across individual IMIDs. Chronotype assessment could contribute to risk stratification in patients with IMIDs. Cross-disciplinary collaboration to understand the role of circadian rhythms and chronotype in driving common inflammatory pathways could help to improve outcomes for patients with IMIDs.

Immune-mediated inflammatory diseases (IMIDs) encompass a broad range of conditions characterized by chronic inflammation leading to tissue damage. Examples of IMIDs include rheumatoid arthritis (RA), inflammatory bowel disease (IBD), psoriasis, asthma, and systemic lupus erythematosus (SLE), which all share common features that facilitate consideration under the umbrella term IMID.

IMIDs significantly cluster, for example, patients with IBD have an increased risk of IMIDs including RA and psoriasis (Bernstein et al., 2005; Burisch et al., 2019). This may be in part due to multiple shared genetic susceptibility loci (Parkes et al., 2013). IMIDs can also involve multiple tissues, for example, patients with psoriasis can develop psoriatic arthritis, patients with IBD can develop extra-intestinal manifestations such as joint or skin inflammation, and patients with SLE can acquire multiple organ inflammation involving the skin, joints, and kidneys, suggesting inflammatory pathways can drive similar damage across multiple organs (Antonelli et al., 2021; Figus et al., 2021; Korman, 2020).

Indeed, the paradigm for grouping IMID pathogenesis by organ is evolving to align with shared molecular inflammatory pathways. Many IMIDs cluster around signature cytokines including interleukin (IL)-23 in IBD and psoriasis, IL-6 in RA, and tumor necrosis factor-α (TNFα) as a common downstream effector in inflammatory arthritis and IBD (Schett et al., 2021). TNFα inhibition with monoclonal antibodies has been a revolutionary treatment for patients with some, but not all, IMIDs, highlighting the importance of cross-specialty mechanistic research. A growing number of cytokines are now therapeutic targets across the IMIDs.

## Circadian Rhythms

Circadian rhythms are repeating 24-h cycles that organize physiological processes to maximize host efficiency and survival. Nearly every cell contains the molecular clockwork machinery required to generate feedback loops that run the biological clock and regulate a multitude of clock-controlled outputs. Internal clock time is set by external stimuli such as light:dark cycles or meal timing. Circadian rhythms regulate immune function including lymphocyte trafficking and a magnitude of inflammatory response (Scheiermann et al., 2018). Experimentally, disruption of circadian rhythms often leads to more severe inflammatory phenotypes, as demonstrated in IMID murine models including RA (Gibbs and Ray, 2013), asthma (Durrington et al., 2020), IBD (Codoñer-Franch and Gombert, 2018), and psoriasis (Ando et al., 2015). Humans with circadian disruption as a result of shift work are at higher risk of IMIDs such as asthma and COVID-19 (Maidstone et al., 2021c, 2021a).

## Chronotype

Chronotype relates to a person’s individual circadian phase and ranges on a scale from morningness to eveningness to give an indication of when key homeostatic processes are most active. Chronotype is shaped by environmental and genetic influences and can be interpreted qualitatively as a personality trait or quantitatively as a phase of entrainment (Jones et al., 2019; Roenneberg, 2015). Phase of entrainment can be calculated with invasive sampling, such as oral swabs for dim light melatonin onset (Lewy et al., 1999) or blood tests for biomarker expression panels (Wittenbrink et al., 2018). However, these costly profiles are harder to perform on large populations. The use of questionnaires such as the Morningness-Eveningness Questionnaire (MEQ) (Horne and Ostberg, 1976) and the Munich Chronotype Questionnaire (MCTQ) (Roenneberg et al., 2003) correlates well with invasive tests and can be completed quickly using either paper or electronic versions to maximize participant recruitment (Kantermann et al., 2015).

The MEQ consists of 19 questions, with higher scores indicating more morningness. The MCTQ collects data on sleep timing during work and work-free days to calculate a midpoint of sleep on free days, corrected for oversleep (MSFsc) (Roenneberg et al., 2012), with earlier midpoint of sleep suggesting a morning preference with earlier phase of entrainment.

Chronotype is a risk factor for cardiometabolic and mental health, with evening types being more susceptible to conditions such as obesity and bipolar disease (Hulsegge et al., 2018; Walsh et al., 2022). Here, we aimed to systematically review the evidence of an association between chronotype and patients with IMIDs.

## Materials and Methods

### Search Terms and Strategy

**Figure 1.**
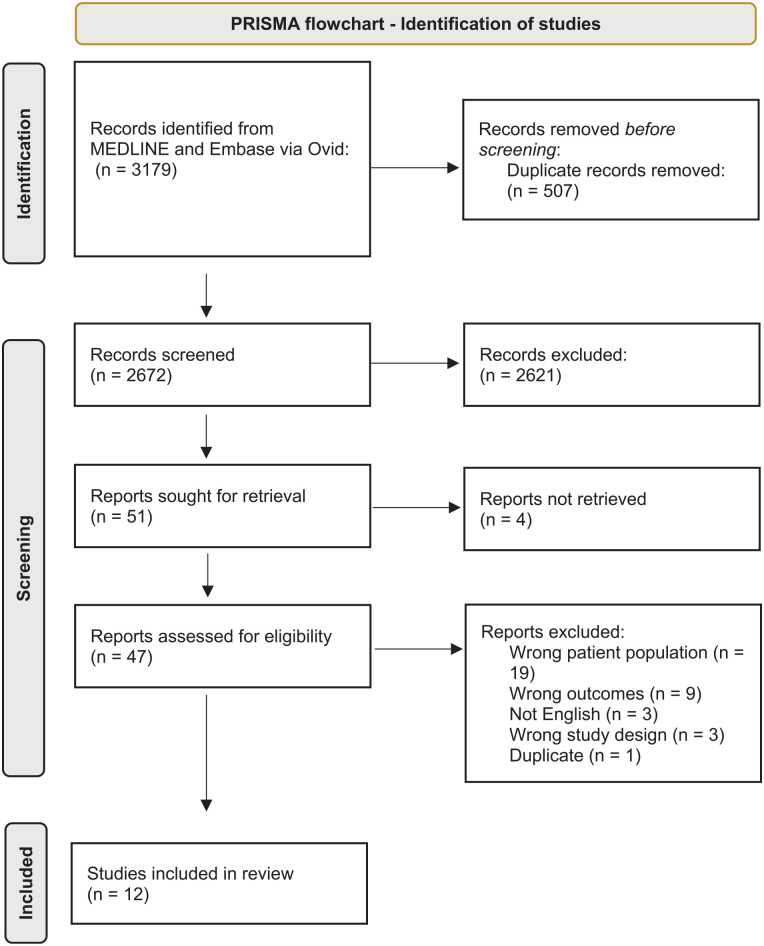
PRISMA flowchart for identification of studies. Abbreviation: Preferred Reporting Items for Systematic Reviews and Meta-Analyses.

This protocol was registered with PROSPERO (CRD42021240942). A comprehensive search was conducted in MEDLINE and Embase, via OVID, to include human studies in English language published between 1 January 2000 and 16 April 2021. The search strategy is included in the appendix and the search process is described as a PRISMA (Preferred Reporting Items for Systematic Reviews and Meta-Analyses) flowchart (Figure 1).

### Eligibility Criteria

Study eligibility was assessed by 2 independent reviewers (T.B. and A.M.A), according to the inclusion and exclusion criteria below:

Inclusion criteria:

(1) Case-control or cross-sectional studies; (2) included patients with RA, IBD, psoriasis, asthma, or SLE; (3) included chronotype data from either MEQ or MCTQ; (4) all clinical settings.

Exclusion criteria:

(1) Study participants <18 years old; (2) review articles.

### Data Extraction and Quality Assessment

Two reviewers (T.B. and A.M.A.) independently examined and extracted the key data using Covidence software. Study quality and risk of bias were assessed by the Newcastle-Ottawa Cohort Scale (NOS) (Wells et al., 2000) and the modified NOS for cross-sectional studies (Herzog et al., 2013). Broadly, the NOS considers studies across 3 domains: study group selection, comparability between groups, and exposure, with the modified NOS considering outcome rather than exposure.

## Results

Twelve studies met the inclusion criteria (Basnet et al., 2018; Chakradeo et al., 2018; Chrobak et al., 2018; Ferraz et al., 2008; Habers et al., 2021; Henry et al., 2017; Keskin and Sahbaz, 2020; Maidstone et al., 2021b; Merikanto et al., 2014a, 2014b; Singh et al., 2016; Wohlfahrt et al., 2014). The included study characteristics are detailed in Table 1 and the main findings are described in Table 2 and Supplementary Table 1. Studies were conducted across 7 countries: the United States (*n* = 3 studies), Finland (*n* = 3), Poland, the Netherlands, Turkey, the United Kingdom, and Brazil (all *n* = 1), with 1 study collecting data internationally. Five studies were classified as case-control and 7 studies were cross-sectional in design. There were no randomized controlled studies or cohort studies. Participants with IMIDs totaled 15,625 with 410,783 healthy controls across all studies (Table 3). Two studies did not have a healthy control (HC) population (Henry et al., 2017; Wohlfahrt et al., 2014) and 1 study did not report numbers of participants with IMID and controls (Basnet et al., 2018). IMIDs included IBD (*n* = 4 studies), RA (*n* = 3), asthma (*n* = 3), both RA and asthma (*n* = 1), and psoriasis (*n* = 1). No relevant papers examining SLE were included.

**Table 1.** Characteristics of studies included in systematic review.

Author (Year)	IMID	Country	Study Design	Study Period	Participants (% Female)	Age [Range], Years	Case Definition	Control Definition	Exclusion Criteria
Singh et al. (2016)	IBD	USA	Case-control study	–	IBD: 123, HC: 76	–	IBD: biopsy-proven	HC	–
Chakradeo et al. (2018)	IBD	USA	Case-control study	–	IBD: 115 (62), HC: 76 (55)	IBD: 34, HC: 41	IBD: endoscopy and biopsy-proven attending Midwestern ambulatory gastroenterology clinic	HC from community with no gastrointestinal conditions	Comorbid disease, HIV, depression, sleep apnea, restless leg syndrome
Chrobak et al. (2018)	IBD	Poland	Case-control study	–	IBD: 72 (53), HC: 57 (56)	IBD: 39 [18-78], HC: 41 [20-67]	IBD: doctor-diagnosed gastroenterology in-patients and out-patients at University Hospital Krakow	HC from community with no self-reported medical conditions	–
Keskin and Sahbaz (2020)	IBD	Turkey	Cross-sectional study	May 2019-September 2019	IBD: 90 (56), HC: 40 (68)	IBD: 37, HC: 40	IBD: endoscopy-proven, attending gastroenterology out-patients	Age-matched HC without chronic disease	Pregnancy, uncertain diagnosis
Merikanto et al. (2014b)	RA	Finland	Cross-sectional study	January 2007-April 2007	RA: 86, HC: 5988	[25-74]	RA: self-reported in FINRISK 2007 study	No RA self-reported	–
Wohlfahrt et al. (2014)	RA	USA	Cross-sectional study	March 2012-June 2012	RA: 191 (85)	RA: 61	RA: RA registry participants with sleep problems (MOS II > 35)	–	–
Habers et al. (2021)	RA	Holland	Case-control study	October 2015	RA: 121 (73)	60 [23-85]	RA: rheumatologist physical examination, attending Leiden early arthritis clinic	Mathematically calculated from Dutch general population database	RA symptoms for longer than 2 years
Basnet et al., (2018)	Asthma and RA	Finland	Cross-sectional study	2012	4414 responses	–	Asthma and RA: self-reported in FINRISK 2012 study	No asthma/RA self-reported	–
Ferraz et al. (2008)	Asthma	Brazil	Case-control study	–	Asthma: 100 (60), HC: 100 (64)	Asthma: 34, HC: 36	Asthma: defined by Global initiative for asthma, attending University Hospital asthma clinic in Brazil	Non-asthmatics who do not co-habit with respiratory patients	–
Merikanto et al. (2014a)	Asthma	Finland	Cross-sectional study	January 2007-April 2007	Asthma: 303, HC: 5644	[25-74]	Asthma: self-reported in FINRISK 2007 study	No self-reported asthma	–
Maidstone et al. (2021b)	Asthma	UK	Cross-sectional study	2007-2010	Asthma: 14,238, HC: 39,8802	[40-69]	Asthma: self-reported doctor-diagnosed and receiving asthma medication	Non-asthmatics	Documented asthma medication without asthma diagnosis
Henry et al. (2017)	Psoriasis	World	Cross-sectional study	June 2015-January 2016	Psoriasis: 186 (75)	Psoriasis: 39 [18-70]	Psoriasis: self-reported doctor-diagnosed plaque psoriasis	-	Age < 18 years; non-English-speaking

Abbreviations: HC = healthy control; IMID = immune-mediated inflammatory disease; MOS = medical outcomes study. Characteristics data from 12 included studies. Dashes (–) represent data not documented or not included.

**Table 2.** Main comparisons, results, and risk of bias assessment for studies included in systematic review.

Author (Year)	IMID	Chronotype	Comparison Made With Chronotype	Main Results	NOS Risk of Bias	Study-Specific Limitations
Singh et al. (2016)	IBD	MEQ, MCTQ	Prevalent IBD and IBD QoL	(1) MEQ: IBD patients had an earlier chronotype (extreme early 15%, intermediate 82%) compared with HC (intermediate 98%)(*p* = 0.003) (2) MEQ: Later chronotype is associated with a lower age-adjusted quality of life (*R* = 0.209, *p* = 0.007) (3) MCTQ: Patients treated with biologics had later MSFsc (3.91 h) compared with patients with no biologic use (3.22 h)(*p* = 0.044)	3/9	Conference abstract, statistical test not documented
Chakradeo et al. (2018)	IBD	MEQ, MCTQ	Prevalent IBD and IBD QoL	(1) MEQ: No difference in mean age-adjusted chronotype between IBD (mean MEQ: 56) and HC (mean MEQ: 53) groups (*p* = 0.27) (2) MCTQ: MSFsc 33 min earlier in IBD vs HC (3.56 ± 1.35 h vs 4.29 ± 1.10 h) (3) MEQ: Correlation between lower SIBDQ QoL and evening chronotype (*R* = −0.209, *p* < 0.05)	6/9	IBD patients grouped increasing heterogeneity in population
Chrobak et al. (2018)	IBD	CSM	IBD-related fatigue	(1) Eveningness associated with increased multidimensional fatigue inventory (MFI) scores (regression coefficients (CD: −0.88; UC: −0.84; *p* < 0.001) (2) CSM did not correlate with IBDQ QoL scores (correlation coefficient: −0.09)	4/9	MFI not validated to determine clinically significant fatigue
Keskin and Sahbaz (2020)	IBD	MEQ	Prevalent IBD and IBD activity	(1) IBD patients had a later chronotype (mean MEQ: 52) compared with HC (mean MEQ: 59) (*p* < 0.001) (2) No difference in chronotype between patients with active and passive IBD (*p* = 0.657) (MEQ scores not documented)	5/10	HCs had average BMI in obese range and significantly more likely to smoke
Merikanto et al. (2014b)	RA	MEQ*	RA incidence and RA symptoms	(1) Intermediate types have higher odds than morning types to have new RA diagnosis in last 12 months (OR 2.0 [95% CI 1.3-3.3]) (2) Compared with morning types, evening types have higher odds of rheumatic symptoms (OR 1.6 [95% CI 1.1 - 2.2]) in the last month (MEQ scores not documented)	9/10	No HC population. RA diagnosis self-reported
Wohlfahrt et al. (2014)	RA	MEQ	RA symptoms	(1) In this cohort, 43 (22.5%) morningness, 67 (35.1%) eveningness, and 81 (42.4% intermediate (2) No difference between morning and evening chronotype and MDHAQ pain (40.6 vs 37.5) and fatigue scales (53.6 vs 53.0)	7/10	Conference abstract, statistical test not documented, no HC group to compare MEQ
Habers et al. (2021)	RA	MCTQ	RA prevalence and RA disease activity	(1) MSFsc was 23 min earlier in RA patients compared with control group (3 h 29 min vs 3 h 52 min, *p* < 0.001) (2) Correlation between RA patients with earlier MSFsc and lower number of swollen joints (*r* = 0.224, *p* = 0.016) (3) No correlation between chronotype and morning stiffness (*p* = 0.444) or fatigue (*p* = 0.415)	9/9	HC comparator group was mathematically calculated; low questionnaire response rate
Basnet et al. (2018)	Asthma and RA	MEQ*	Prevalence of RA or asthma	(1) Evening types had lower odds of bronchial asthma compared with morning types (OR 0.46 [95% CI 0.27-0.77]) (2) No difference in odds of RA between evening and morning types (OR 1.19 [95% CI 0.47-3.00])	9/10	Asthma and RA diagnoses self-reported. Counts of case frequency not documented
Ferraz et al. (2008)	Asthma	MEQ	Asthma prevalence and disease activity	(1) No difference in chronotype distribution between asthmatics (mean MEQ: 58) and HC (mean MEQ: 60) (*p* = 0.15) (2) Asthmatics with nocturnal symptoms were more frequently intermediate chronotype (*p* < 0.05) and less frequently “moderately morning” chronotype (*p* < 0.05), compared with asthmatics without nocturnal symptoms (MEQ scores not documented)	5/9	Score thresholds to categorize MEQ chronotypes are different in Brazil
Merikanto et al. (2014a)	Asthma	MEQ*	Asthma prevalence and asthmatic symptoms	(1) Evening types had higher odds of asthma, compared with morning types (OR 1.9 [95% CI 1.3-2.7]) (2) Compared with morning types, evening types have higher odds of nocturnal breathlessness (OR 1.8 [95% CI 1.3-2.5]) and nocturnal cough (OR 1.4 [95% CI 1.2-1.7]) (MEQ scores not documented)	9/10	Self-reported asthma: nocturnal breathlessness and cough have broad differential
Maidstone et al. (2021b)	Asthma	MEQ*	Asthma prevalence	(1) Compared with intermediate chronotypes, extreme morning types (OR 1.12 [95% CI 1.03-1.21]) and extreme evening types (OR 1.16 [95% CI 1.04-1.28]) had higher odds of asthma (MEQ scores not documented)	10/10	Asthma was self-reported (but also required an asthma medication)
Henry et al. (2017)	Psoriasis	MEQ	Psoriasis prevalence	(1) MEQ: Mean MEQ in psoriasis patients = 51	4/10	No HC comparator, self-reported psoriasis; low response rate; study population 75% female

Abbreviations: IMID = Immune-mediated inflammatory diseases; NOS = Newcastle-Ottawa Cohort Scale; IBD = inflammatory bowel disease; MEQ = Morningness-Eveningness Questionnaire; MEQ* = abridged MEQ; MCTQ = Munich Chronotype Questionnaire; HC = healthy control; SIBDQ = Short Inflammatory Bowel Disease Questionnaire; CSM = Composite Scale Of Morningness; MFI = Multidimensional Fatigue Inventory; CD = Crohn’s disease; UC = ulcerative colitis; MDHAQ = multidimensional health assessment questionnaire; BMI = body mass index; RA = rheumatoid arthritis; OR = odds ratio; CI = confidence interval; MSFsc = corrected midpoint of sleep; QoL = quality of life. Data from the 12 included studies. NOS for risk of bias graded out of 9 for case-control studies and out of 10 for cross-sectional studies. A higher NOS score represents lower risk of bias.

**Table 3.** The number of participants included in studies.

IMID	No. of Participants
IBD	400
RA	398
Asthma	14,641^a^
Psoriasis	186
Healthy control	410,783^b^

Abbreviations: IMID = Immune-mediated inflammatory diseases; IBD = inflammatory bowel disease; RA = rheumatoid arthritis. The number of participants included for each IMID condition and healthy controls.

a Number of participants with asthma not documented in 1 study (Basnet et al., 2018).

b Number of healthy controls not documented in 1 study. Note that healthy controls were not used in every study.

Case definition of included IMID patients ranged from self-report of doctor-diagnosed condition to confirmed clinical diagnosis by specialist doctor at secondary care clinics. Four studies independently verified case definitions with data from primary records (Chakradeo et al., 2018; Ferraz et al., 2008; Habers et al., 2021; Singh et al., 2016). Where HCs were used in case-control studies, individuals were identified in the local community with no self-reported IMIDs. In the majority of cross-sectional studies, HCs were identified by the absence of self-reported IMID being studied. One study mathematically calculated HC data by taking the MSFsc values from a large Dutch database and constructing a best fit line used to extrapolate age- and gender-matched data (Habers et al., 2021).

Comparability of cases and controls was considered to varying degrees across studies (Table 4). Maidstone et al. employed 3 detailed models (Maidstone et al., 2021b), whereas 4 studies did not document any methods for matching or adjusting data (Chrobak et al., 2018; Ferraz et al., 2008; Habers et al., 2021; Henry et al., 2017). The heterogeneity of reported data only permitted descriptive statistics. Data to perform a meta-analysis of effect estimates were unavailable.

**Table 4.** List of covariates used by included studies.

Author (Year)	Adjustment/Matching
Singh et al. (2016)	Age
Chakradeo et al. (2018)	Age
Chrobak et al. (2018)	—
Keskin and Sahbaz (2020)	Age-matched HC
Merikanto et al. (2014a)	Model 1: crude; model 2: age and gender; model 3: model 2 + education level, civil status, physical activity, alcohol consumption, current smoking
Wohlfahrt et al. (2014)	Multivariable linear regression model: age, sex, serology, disease activity (DAS28-CRP)
Habers et al. (2021)	—
Mannisto et al. (2018)	BMI, age, sex, civil status, education level, region, smoking alcohol consumption, physical activity
Ferraz et al. (2008)	—
Merikanto et al. (2014b)	Age, sex, education level, civil status, physical activity, alcohol use, smoking
Maidstone et al. (2021b)	Model 1: age and sex; model 2: model 1 plus BMI, ethnicity, chronotype, TDI, days exercised, smoker status and pack-years smoked, alcohol status and alcohol weekly intake, length of working week, occupational asthma risk; model 3: model 2 plus sleep deprivation
Henry et al. (2017)	—

Abbreviations: DAS = Disease activity score; BMI = body mass index; HC = healthy controls; TDI = Townsend Deprivation Index.

Four studies compared MEQ chronotype in IMID versus HC (Chakradeo et al., 2018; Ferraz et al., 2008; Keskin and Sahbaz, 2020; Singh et al., 2016). In 1 study, an earlier chronotype was more likely in IMID patients compared with HC (IBD), 2 studies found no difference in chronotype (IBD and asthma), and 1 study identified a later chronotype in IMID patients (IBD). Two of 2 (100%) studies with MCTQ data reported an earlier MSFsc in IMID patients compared with HC (Chakradeo et al., 2018; Habers et al., 2021).

Four studies examined the association of chronotype with odds of disease (Basnet et al., 2018; Maidstone et al., 2021b; Merikanto et al., 2014a, 2014b). No trends were seen across the studies, which used different comparators. For example, the three asthma studies demonstrated that evening types had higher odds of asthma compared with morning types (Merikanto et al., 2014a), lower odds of asthma compared with morning types (Basnet et al., 2018), and that extremes of chronotype have more asthma than intermediate types (Maidstone et al., 2021b).

Seven studies investigated the impact of chronotype on IMID quality of life and symptom burden (Chakradeo et al., 2018; Chrobak et al., 2018; Habers et al., 2021; Merikanto et al., 2014a, 2014b; Singh et al., 2016; Wohlfahrt et al., 2014). Five of 7 (71%) studies reported significantly worse morbidity in patients with later chronotypes, using validated metrics such as the Short IBD Questionnaire (SIBDQ), as well as patient-reported symptom burden. Two studies reported no difference in disease activity (IBD) or fatigue and pain (RA).

## Discussion

This systematic review of 12 papers relating to the role of chronotype in IMID has revealed that a later chronotype may be a risk factor for worse quality of life and increased symptom burden across patients with IMIDs. The review also suggests that chronotype may be a risk factor for IMID, but the direction and magnitude of this effect vary between studies and IMID conditions.

The presence of a later chronotype associating with morbidity was interestingly observed in patients with asthma, RA, and IBD. Asthma and RA have clear diurnal variation in symptoms, with asthma exacerbations worse at night and RA joint stiffness and pain worse in the morning, correlating with peak rhythmic proinflammatory cytokine expression (Gibbs and Ray, 2013). In addition, IBD does not have a clear diurnal variation in symptoms and can present with nocturnal diarrhea, which may represent a loss of rhythmicity in homeostatic defecation. Together, this suggests that later chronotypes may be less able to effectively gate immune responses and suppress inflammatory cascades, regardless of the IMID’s peak inflammatory period. This encourages further investigation of circadian influence on common IMID inflammatory pathways.

Studies of MEQ chronotype did not demonstrate a predominant trend for chronotype in IMID. Where the mean MEQ was available, this fell within the intermediate category for IMIDs and HC, suggesting that statistically significant differences may only have limited biological significance. Two studies of MCTQ chronotype demonstrated an earlier MSFsc by 23-33 min in RA and IBD, respectively. This could suggest that phase of entrainment measured by MCTQ is more relevant than chronotype as a personality trait measured by MEQ. Regardless, further study is required to robustly characterize chronotype across a multitude of IMIDs, with reproducible and consistent metrics.

This study contributes to the growing evidence that chronotype is a relevant factor for disease development and progression. As evidence for the association of chronotype with inflammatory disease grows and the molecular drivers are investigated, the use of objective chronotype measures becomes more relevant as a method of potentially reducing bias that arises from self-reported questionnaire-based chronotype assessments. For example, blood tests such as BodyTime use biomarkers to calculate phase, with similar success in skin biopsies (Wittenbrink et al., 2018; Anafi et al., 2017). These more accurate measures of phase of entrainment could influence personalized chronotherapy by aligning medication administration to chronotype for improved efficacy.

The requirement for research into patients with IMIDs and novel insights into shared pathogenesis have a good prospect of progression with the help of IMID databases and biobanks, such as the National Institute for Health and Care Research (NIHR) IMID BioResource and IMID-Bio-UK biobanks; however, it is unclear whether chronotype is an included assessment. It is important to continue to investigate and understand common as well as unique pathways across IMID conditions with the aim of improving patient care through identification of diagnostic biomarkers and therapeutic advances such as prediction of response to therapy. Investigation of genetics across IMIDs provides an example of the rewards and challenges of identifying commonality across inflammatory diseases. Immunochip single nucleotide polymorphism (SNP) microarray technology has revealed 71 loci that significantly associate with 2 or more IMIDs; however, 14% of hits were discordant, meaning their modification of risk was increased in some IMIDs and down in others (Parkes et al., 2013). Of interest, genetic studies have highlighted components of the IL-23R response pathway significantly associated with a broad range of IMIDs, leading to novel understanding of the role of IL-23 in IMID pathogenesis, and contributed to the discovery of therapies targeting IL-23, currently licensed for use in psoriasis and IBD in the United Kingdom (Bianchi and Rogge, 2019). The circadian clock regulates components of this pathway, including Th17 effector cells and their innate counterparts, type 3 innate lymphoid cells (Yu et al., 2013; Butler and Gibbs, 2020; Godinho-Silva et al., 2019). Further work is required to explore the regulatory role of circadian rhythms in the IL-23 pathway across different inflammatory disease models.

To our knowledge, this is the first systematic review of multiple IMIDs and the contribution of chronotype. A broad search strategy covering over 20 years of research identified a collection of papers that can be taken together to understand the role of chronotype preference in chronic inflammatory conditions. Collating these studies across different diseases will help to guide future work. This work does carry limitations, including the heterogeneity of data representation and comparator groups across studies. Raw chronotype data were not available from any included study, precluding quantitative meta-analysis. Many studies did not clearly define cases and lacked adjustment for common variables known to influence chronotype, such as age and sex, making it harder to draw comparative conclusions.

## Conclusion

IMIDs are a collection of chronic, often debilitating inflammatory diseases managed by separate clinical specialties. A patient’s chronotype, easily calculated via questionnaire, may help to predict the severity of symptoms in IMIDs and could contribute to risk stratification for monitoring and treatment purposes. It must be recognized that IMID patient groups can vary by age, sex, and ethnicity, requiring a patient-centered multidisciplinary treatment approach rather than a one-size-fits-all strategy. However, there is growing evidence of commonality in pathology across IMIDs at a genetic, cellular, and environmental level. The trend for evening chronotypes to experience more serious symptoms across different IMIDs highlights the potential role of circadian rhythms in gating common inflammatory pathways in IMIDs. Further research can now focus on developing strategies for robust cross-disciplinary circadian research in the hope of furthering IMID management.

## Supplemental Material

sj-xlsx-1-jbr-10.1177_07487304221131114 – Supplemental material for Chronotype in Patients With Immune-Mediated Inflammatory Disease: A Systematic Review Supplemental material, sj-xlsx-1-jbr-10.1177_07487304221131114 for Chronotype in Patients With Immune-Mediated Inflammatory Disease: A Systematic Review by Thomas D. Butler, Aala Mohammed Ali, Julie E. Gibbs and John T. McLaughlin in Journal of Biological Rhythms

## Appendix

**Appendix.** Search strategy for Ovid MEDLINE and Epub Ahead of Print, In-Process, In-Data-Review & Other Non-Indexed Citations and Daily, 1946-present, Embase 1974-present, and EBM Reviews—Cochrane Database of Systematic Reviews 2005-present.

Ovid MEDLINE(R) and Epub Ahead of Print, In-Process, In-Data-Review & Other Non-Indexed Citations, Daily and Versions <1946 to 6 May 2022>	
Embase <1974 to 6 May 2022>	
EBM Reviews—Cochrane Database of Systematic Reviews <2005 to 5 May 2022>	
1.	chrono*.mp. [mp=ti, ot, ab, tx, kw, ct, sh, hw, tn, dm, mf, dv, kf, fx, dq, nm, ox, px, rx, an, ui, sy]
2.	munich chrono* question*.mp. [mp=ti, ot, ab, tx, kw, ct, sh, hw, tn, dm, mf, dv, kf, fx, dq, nm, ox, px, rx, an, ui, sy]
3.	morningness.mp. [mp=ti, ot, ab, tx, kw, ct, sh, hw, tn, dm, mf, dv, kf, fx, dq, nm, ox, px, rx, an, ui, sy]
4.	inflam*.mp. [mp=ti, ot, ab, tx, kw, ct, sh, hw, tn, dm, mf, dv, kf, fx, dq, nm, ox, px, rx, an, ui, sy]
5.	autoimmun*.mp. [mp=ti, ot, ab, tx, kw, ct, sh, hw, tn, dm, mf, dv, kf, fx, dq, nm, ox, px, rx, an, ui, sy]
6.	psoria*.mp. [mp=ti, ot, ab, tx, kw, ct, sh, hw, tn, dm, mf, dv, kf, fx, dq, nm, ox, px, rx, an, ui, sy]
7.	asthm*.mp. [mp=ti, ot, ab, tx, kw, ct, sh, hw, tn, dm, mf, dv, kf, fx, dq, nm, ox, px, rx, an, ui, sy]
8.	inflammatory bowel diseas*.mp. [mp=ti, ot, ab, tx, kw, ct, sh, hw, tn, dm, mf, dv, kf, fx, dq, nm, ox, px, rx, an, ui, sy]
9.	ulcerative colitis.mp. [mp=ti, ot, ab, tx, kw, ct, sh, hw, tn, dm, mf, dv, kf, fx, dq, nm, ox, px, rx, an, ui, sy]
10.	crohn* disease.mp. [mp=ti, ot, ab, tx, kw, ct, sh, hw, tn, dm, mf, dv, kf, fx, dq, nm, ox, px, rx, an, ui, sy]
11.	rheumatoid arthritis.mp. [mp=ti, ot, ab, tx, kw, ct, sh, hw, tn, dm, mf, dv, kf, fx, dq, nm, ox, px, rx, an, ui, sy]
12.	sle.mp. [mp=ti, ot, ab, tx, kw, ct, sh, hw, tn, dm, mf, dv, kf, fx, dq, nm, ox, px, rx, an, ui, sy]
13.	systemic lupus erythematosus.mp. [mp=ti, ot, ab, tx, kw, ct, sh, hw, tn, dm, mf, dv, kf, fx, dq, nm, ox, px, rx, an, ui, sy]
14.	1 or 2 or 3
15.	4 or 5 or 6 or 7 or 8 or 9 or 10 or 11 or 12 or 13
16.	14 and 15
17.	limit 16 to humans
18.	limit 17 to yr =“2000 -Current”
19.	“review”.mp. [mp=ti, ot, ab, tx, kw, ct, sh, hw, tn, dm, mf, dv, kf, fx, dq, nm, ox, px, rx, an, ui, sy]
20.	18 not 19

## References

[bibr1-07487304221131114] Anafi RC Francey LJ Hogenesch JB Kim J (2017) CYCLOPS reveals human transcriptional rhythms in health and disease. Proc Natl Acad Sci USA 114:5312-5317. 28439010 10.1073/pnas.1619320114 PMC5441789

[bibr2-07487304221131114] Ando N Nakamura Y Aoki R Ishimaru K Ogawa H Okumura K Shibata S Shimada S Nakao A (2015) Circadian gene clock regulates psoriasis-like skin inflammation in mice. J Invest Dermatol 135:3001-3008. 26291684 10.1038/jid.2015.316 PMC4653315

[bibr3-07487304221131114] Antonelli E Bassotti G Tramontana M Hansel K Stingeni L Ardizzone S Genovese G Marzano AV Maconi G (2021) Dermatological manifestations in inflammatory Bowel diseases. J Clin Med 10:364. 33477990 10.3390/jcm10020364 PMC7835974

[bibr4-07487304221131114] Basnet S Merikanto I Lahti T Männistö S Laatikainen T Vartiainen E Partonen T (2018) Seasonality, morningness–eveningness, and sleep in common non-communicable medical conditions and chronic diseases in a population. Sleep Sci 11:85-91. 30083295 10.5935/1984-0063.20180017 PMC6056070

[bibr5-07487304221131114] Bernstein CN Wajda A Blanchard JF (2005) The clustering of other chronic inflammatory diseases in inflammatory Bowel disease: a population-based study. Gastroenterology 129:827-836. 16143122 10.1053/j.gastro.2005.06.021

[bibr6-07487304221131114] Bianchi E Rogge L (2019) The IL-23/IL-17 pathway in human chronic inflammatory diseases—new insight from genetics and targeted therapies. Genes Immun 20:415-425. 31000797 10.1038/s41435-019-0067-y

[bibr7-07487304221131114] Burisch J Jess T Egeberg A (2019) Incidence of immune-mediated inflammatory diseases among patients with inflammatory Bowel diseases in Denmark. Clin Gastroenterol Hepatol 17:2704-2712e2703. 10.1016/j.cgh.2019.03.040 30936024

[bibr8-07487304221131114] Butler TD Gibbs JE (2020) Circadian host-microbiome interactions in immunity. Front Immunol 11:1783. 10.3389/fimmu.2020.01783 PMC7456996 32922391

[bibr9-07487304221131114] Chakradeo PS Keshavarzian A Singh S Dera AE Esteban JPG Lee AA Burgess HJ Fogg L Swanson GR (2018) Chronotype, social jet lag, sleep debt and food timing in inflammatory bowel disease. Sleep Med 52:188-195. 30243610 10.1016/j.sleep.2018.08.002 PMC8177729

[bibr10-07487304221131114] Chrobak AA Nowakowski J Zwolińska-Wcisło M Cibor D Przybylska-Feluś M Ochyra K Rzeźnik M Dudek A Arciszewska A Siwek M , et al. (2018) Associations between chronotype, sleep disturbances and seasonality with fatigue and inflammatory bowel disease symptoms. Chronobiol Int 35:1142-1152. 29737879 10.1080/07420528.2018.1463236

[bibr11-07487304221131114] Codoñer-Franch P Gombert M (2018) Circadian rhythms in the pathogenesis of gastrointestinal diseases. World J Gastroenterol 24:4297-4303. 30344415 10.3748/wjg.v24.i38.4297 PMC6189841

[bibr12-07487304221131114] Durrington HJ Krakowiak K Meijer P Begley N Maidstone R Goosey L Gibbs JE Blaikley JF Gregory LG Lloyd CM , et al. (2020) Circadian asthma airway responses are gated by REV-ERBα. Eur Respir J 56:1902407. 32586876 10.1183/13993003.02407-2019 PMC7613655

[bibr13-07487304221131114] Ferraz E Borges MC Vianna EO (2008) Influence of nocturnal asthma on chronotype. J Asthma 45:911-915. 19085582 10.1080/02770900802395470

[bibr14-07487304221131114] Figus FA Piga M Azzolin I McConnell R Iagnocco A (2021) Rheumatoid arthritis: extra-articular manifestations and comorbidities. Autoimmun Rev 20:102776. 33609792 10.1016/j.autrev.2021.102776

[bibr15-07487304221131114] Gibbs JE Ray DW (2013) The role of the circadian clock in rheumatoid arthritis. Arthritis Res Ther 15:205. 23427807 10.1186/ar4146 PMC3672712

[bibr16-07487304221131114] Godinho-Silva C Domingues RG Rendas M Raposo B Ribeiro H da Silva JA Vieira A Costa RM Barbosa-Morais NL Carvalho T , et al. (2019) Light-entrained and brain-tuned circadian circuits regulate ILC3s and gut homeostasis. Nature 574:254-258. 31534216 10.1038/s41586-019-1579-3 PMC6788927

[bibr17-07487304221131114] Habers GEA van der Helm-van Mil AHM Veldhuijzen DS Allaart CF Vreugdenhil E Starreveld DEJ Huizinga TWJ Evers AWM (2021) Earlier chronotype in patients with rheumatoid arthritis. Clin Rheumatol 40:2185-2192. 33452937 10.1007/s10067-020-05546-x PMC8121723

[bibr18-07487304221131114] Henry AL Kyle SD Chisholm A Griffiths CEM Bundy C (2017) A cross-sectional survey of the nature and correlates of sleep disturbance in people with psoriasis. Br J Dermatol 177:1052-1059. 28314054 10.1111/bjd.15469

[bibr19-07487304221131114] Herzog R Álvarez-Pasquin MJ Díaz C Del Barrio JL Estrada JM Gil Á (2013) Are healthcare workers’ intentions to vaccinate related to their knowledge, beliefs and attitudes? a systematic review. BMC Public Health 13:154. 23421987 10.1186/1471-2458-13-154 PMC3602084

[bibr20-07487304221131114] Horne JA Ostberg O (1976) A self-assessment questionnaire to determine morningness-eveningness in human circadian rhythms. Int J Chronobiol 4:97-110. 1027738

[bibr21-07487304221131114] Hulsegge G Picavet HSJ van der Beek AJ Verschuren WMM Twisk JW Proper KI (2018) Shift work, chronotype and the risk of cardiometabolic risk factors. Eur J Public Health 29:128-134. 10.1093/eurpub/cky092 29796606

[bibr22-07487304221131114] Jones SE Lane JM Wood AR van Hees VT Tyrrell J Beaumont RN Jeffries AR Dashti HS Hillsdon M Ruth KS , et al. (2019) Genome-wide association analyses of chronotype in 697,828 individuals provides insights into circadian rhythms. Nat Commun 10:343. 30696823 10.1038/s41467-018-08259-7 PMC6351539

[bibr23-07487304221131114] Kantermann T Sung H Burgess HJ (2015) Comparing the morningness-eveningness questionnaire and munich chronotype questionnaire to the dim light melatonin onset. J Biol Rhythms 30:449-453. 26243627 10.1177/0748730415597520 PMC4580371

[bibr24-07487304221131114] Keskin EB Sahbaz C (2020) Chronotype and sleep quality in patients with inflammatory bowel disease. Med Bull Haseki 58:72-77.

[bibr25-07487304221131114] Korman NJ (2020) Management of psoriasis as a systemic disease: what is the evidence? Br J Dermatol 182:840-848. 31225638 10.1111/bjd.18245 PMC7187293

[bibr26-07487304221131114] Lewy AJ Cutler NL Sack RL (1999) The endogenous melatonin profile as a marker for circadian phase position. Journal of Biological Rhythms 14:227-236. 10452335 10.1177/074873099129000641

[bibr27-07487304221131114] Maidstone RJ Anderson SG Ray DW Rutter MK Durrington HJ Blaikley JF (2021a) Shift work is associated with positive COVID-19 status in hospitalised patients. Thorax 76:601-606. 33903187 10.1136/thoraxjnl-2020-216651 PMC8098298

[bibr28-07487304221131114] Maidstone RJ Turner J Vetter C Dashti HS Saxena R Scheer F Shea SA Kyle SD Lawlor DA Loudon ASI , et al. (2021b) Night shift work is associated with an increased risk of asthma. Thorax 76:53-60. 33199525 10.1136/thoraxjnl-2020-215218 PMC7803886

[bibr29-07487304221131114] Maidstone RJ Turner J Vetter C Dashti HS Saxena R Scheer FAJL Shea SA Kyle SD Lawlor DA Loudon ASI Blaikley JF , et al. (2021c) Night shift work is associated with an increased risk of asthma. Thorax 76:53. 33199525 10.1136/thoraxjnl-2020-215218 PMC7803886

[bibr30-07487304221131114] Merikanto I Englund A Kronholm E Laatikainen T Peltonen M Vartiainen E Partonen T (2014a) Evening chronotypes have the increased odds for bronchial asthma and nocturnal asthma. Chronobiol Int 31:95-101. 24131153 10.3109/07420528.2013.826672

[bibr31-07487304221131114] Merikanto I Lahti T Seitsalo S Kronholm E Laatikainen T Peltonen M Vartiainen E Partonen T (2014b) Behavioral trait of morningness-eveningness in association with articular and spinal diseases in a population. PLoS ONE 9:e114635. 10.1371/journal.pone.0114635 PMC4255027 25470493

[bibr32-07487304221131114] Parkes M Cortes A van Heel DA Brown MA (2013) Genetic insights into common pathways and complex relationships among immune-mediated diseases. Nat Rev Genet 14:661-673. 23917628 10.1038/nrg3502

[bibr33-07487304221131114] Roenneberg T (2015) Having trouble typing? What on earth is chronotype? J Biol Rhythms 30:487-491. 26446872 10.1177/0748730415603835

[bibr34-07487304221131114] Roenneberg T Allebrandt KV Merrow M Vetter C (2012) Social jetlag and obesity. Curr Biol 22:939-943. 22578422 10.1016/j.cub.2012.03.038

[bibr35-07487304221131114] Roenneberg T Wirz-Justice A Merrow M (2003) Life between clocks: daily temporal patterns of human chronotypes. J Biol Rhythms 18:80-90. 12568247 10.1177/0748730402239679

[bibr36-07487304221131114] Scheiermann C Gibbs J Ince L Loudon A (2018) Clocking in to immunity. Nat Rev Immunol 18:423-437. 29662121 10.1038/s41577-018-0008-4

[bibr37-07487304221131114] Schett G McInnes IB Neurath MF (2021) Reframing immune-mediated inflammatory diseases through signature cytokine hubs. N Engl J Med 385:628-639. 34379924 10.1056/NEJMra1909094

[bibr38-07487304221131114] Singh S Dera AE Esteban J Chakradeo PS Lee A Burgess HR Keshavarzian A Swanson GR (2016) Tu1992 Later chronotype is associated with worse quality of life and biologic use in inflammatory bowel disease. Gastroenterology 150:S999-S1000.

[bibr39-07487304221131114] Walsh NA Repa LM Garland SN (2022) Mindful larks and lonely owls: the relationship between chronotype, mental health, sleep quality, and social support in young adults. J Sleep Res 31:e13442. 10.1111/jsr.13442 34272788

[bibr40-07487304221131114] Wells GA Shea B O’Connell D Peterson J Welch V Losos M Tugwell P (2000) The Newcastle-ottawa scale (NOS) for assessing the quality of nonrandomised studies in meta-analyses. https://www.ohri.ca/programs/clinical_epidemiology/oxford.asp.

[bibr41-07487304221131114] Wittenbrink N Ananthasubramaniam B Münch M Koller B Maier B Weschke C Bes F de Zeeuw J Nowozin C Wahnschaffe A , et al. (2018) High-accuracy determination of internal circadian time from a single blood sample. J Clin Invest 128:3826-3839. 29953415 10.1172/JCI120874 PMC6118629

[bibr42-07487304221131114] Wohlfahrt ACJ Frits MA Iannaccone CK Coblyn JS Weinblatt ME Shadick NA Lee YC (2014) Early birds versus night owls: morning/evening preference and its association with sleep problems, fatigue, and emotional well-being among RA patients. In: 2014 ACR/ARHP Annual Meeting Abstract 1335. https://acrabstracts.org/abstract/early-birds-versus-night-owls-morningevening-preference-and-its-association-with-sleep-problems-fatigue-and-emotional-well-being-among-ra-patients/.

[bibr43-07487304221131114] Yu X Rollins D Ruhn KA Stubblefield JJ Green CB Kashiwada M Rothman PB Takahashi JS Hooper LV (2013) TH17 cell differentiation is regulated by the circadian clock. Science 342:727-730. 24202171 10.1126/science.1243884 PMC4165400

